# Computed tomography of pediatric abdominal trauma: optimizing utilization and enhancing diagnostic interpretation

**DOI:** 10.1007/s00247-025-06321-3

**Published:** 2025-07-18

**Authors:** Shaimaa AbdelSattar Mohammad, Reem Abdelhamed Elsayed, Nouran Esam Arafa, Esraa Yahia Ragab, Nesma Naguib Elsayed, Mirna Eehab Hassan, Marwa Mohammad Abdulghaffar, Ghada Samir Ibrahim

**Affiliations:** 1 Department of diagnostic and interventional radiology and molecular imaging, Ain Shams University, Cairo, Egypt; 2 Pediatric Radiology section, Department of diagnostic and interventional radiology and molecular imaging, Ain Shams University, Cairo, 1181 Egypt

**Keywords:** Blunt abdominal injury, Gall bladder, Gastrointestinal perforation, Hemoperitoneum, Kidney trauma, Liver contusion, Radiation exposure

## Abstract

Abdominal trauma is the leading cause of fatal injuries in pediatric patients, affecting approximately 25% of children with major trauma. Computed tomography (CT) remains the gold standard imaging modality for assessing stable patients with potential abdominal injuries. This review outlines CT protocols specifically adapted for the pediatric population in the context of blunt abdominal trauma, with a focus on strategies to mitigate radiation exposure. It also highlights the 2018 update of the American Association for the Surgery of Trauma Organ Injury Scale—a detailed classification system ranging from grade I (minor) to grade V (severe)—and explores its relevance and impact on the management of pediatric patients compared to adults. A series of CT images illustrating the different grades of abdominal injuries along with a structured interpretation algorithm are provided to act as a pocket guide for radiology residents in emergency settings.

**Graphical Abstract:**

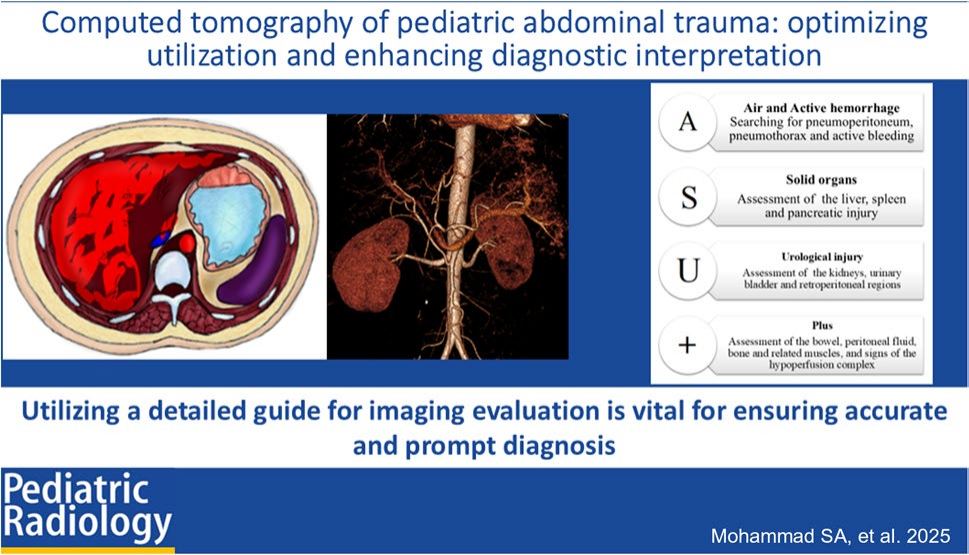

**Supplementary Information:**

The online version contains supplementary material available at 10.1007/s00247-025-06321-3.

## Introduction

Trauma is among the leading causes of mortality in pediatric patients [1]. Abdominal injury affects approximately 25% of children who have experienced major trauma [2]. Abdominal trauma in children is the third leading cause of death after head and thoracic injuries [3]. Compared to adults, children are more vulnerable to blunt abdominal injury because of their smaller abdominal cavity, thinner visceral and subcutaneous fat, relatively larger organs, and weaker muscles [4, 5].

After stabilization and identification of any life-threatening injuries in accordance with the protocols of Advanced Trauma Life Support, Extended Focused Assessment with Sonography for Trauma (EFAST) is the initial imaging test employed in the evaluation of abdominal trauma patients [6, 7]. However, the EFAST exam was found to have poor sensitivity in detecting abdominal injuries in children. It can miss serious injury and should be used with caution [8–13]. Its role may be most obvious in hemodynamically unstable children, as it may detect bloody collections in the pleura, peritoneum, and pericardium which significantly identify the need for early surgical intervention promptly [13–15].

Computed tomography (CT) is the best imaging modality in hemodynamically stable patients with abdominal injuries [16–20]. Children are more susceptible to radiation exposure than adults due to a smaller body size, more dividing cells, and a longer life span to manifest potential radiation hazards [21, 22]. A large multicenter study with individualized radiation dose assessments demonstrated a clear dose-dependent association between CT exposure and brain cancer risk in children, underlining the importance of rigorously justifying pediatric CT use and minimizing radiation doses in accordance with the As Low As Reasonably Achievable principle [23]. Various professional societies and organizations developed algorithms and clinical prediction rules to guide the use of radiographic imaging following traumatic injuries. These algorithms rely on medical history and abdominal clinical assessment, and may incorporate laboratory tests as aspartate aminotransferase, lipase, and urine analysis [24–30]. A summary of common imaging algorithms is provided in Table 1.

**Table 1:** Pediatric blunt abdominal trauma imaging algorithms

Algorithm	Parameters		Remarks
The blunt abdominal trauma in children (BATiC) [26]	Abnormal abdominal Doppler US Abdominal pain Signs of peritoneal irritation Hemodynamic instability AST>60 IU/L ALT>25 IU/L WBC count>9.5 g/L LDH>330 IU/L Lipase 30 IU/L Creatinine>50 µg/L	4 points 2 points 2 points 2 points 2points 2 points 1 point 1point 1 point 1 point	BATiC score is calculated by summing the points for each parameter ≤ 7 renders intra-abdominal injury unlikely in hemodynamically stable patients and CT may be avoided The score range is 0 to 18
Pediatric Emergency Care Applied Research Network (PECARN) prediction rule of 7 [27]	No evidence of abdominal wall trauma or evidence of seat belt sign Glasgow Coma Scale score greater than 13 No abdominal tenderness No evidence of thoracic wall trauma No complaints of abdominal pain No decreased breath sounds No vomiting		This prediction rule of seven history and clinical examination parameters (without laboratory or ultrasound findings) identifies patients who are at very low risk for intra-abdominal injury
Pediatric Surgery Research Collaborative [29]	AST>200 U/L Abnormal abdominal examination Abnormal chest radiograph Report of abdominal pain Abnormal pancreatic enzymes		The rule had a negative predictive value of 99.4% for intra-abdominal injury and 100.0% for intra-abdominal injury requiring intervention in patients with none of the prediction rule variables present
Blunt pediatric abdominal trauma guideline [30]	Abnormal FAST necessitates CT If the ultrasound is normal, AST<200, ALT<100, Hgb>8.5, and urine analysis<0–5 RBCs/HPF, observe for 12–24 h. Otherwise, CT is indicated During observation, if the serial abdominal exams are normal with normal WBC, Hgb drop less than 1.5, and temperature < 101 F, the patient can be discharged without CT. Otherwise, CT is indicated		The application of an evidence-based clinical guideline reduces the frequency of CT use while precisely detecting serious injuries

*ALT* alanine aminotransferase, *AST* aspartate aminotransferase, *BATiC* the blunt abdominal trauma in children, *CT* computed tomography, *FAST* focused assessment with sonography in trauma, *Hgb* hemoglobin, *HPF* high power field, *LDH* lactate dehydrogenase, *PECARN* Pediatric Emergency Care Applied Research Network, *RBC* red blood corpuscles, *US* ultrasound, *WBC* white blood cells

Magnetic resonance imaging (MRI) offers a highly detailed anatomical resolution without radiation exposure, making it a valuable complement to CT [31]. However, its use in the initial evaluation of acute trauma is limited due to restricted availability, longer scanning times, numerous contraindications, and increased technical demands compared to CT. MR imaging is typically performed after an initial CT scan when further evaluation is needed, particularly for complex craniospinal and musculoskeletal soft tissue injuries [32]. MRI may be a suitable alternative to CT in stable patients with severe contrast allergy and acute renal failure, or when exposure to radiation should be avoided (e.g., during pregnancy). MRI is valuable in evaluating trauma to the pancreas and hepatobiliary and genitourinary systems and for detecting diaphragmatic injuries [31–33]. Opting for MRI in follow-up imaging instead of CT is essential for minimizing radiation exposure. MRI may serve as a problem-solving modality in questionable CT findings (e.g., differentiation of fatty liver infiltration versus contusion) or when the CT study is suboptimal (due to upper limb positioning) [31]. MRI protocols should be customized for each patient. Only the most essential sequences should be used to reduce scan time. Free-breathing or respiratory-triggered techniques are preferred especially for patients unable to hold their breath or follow instructions. Diffusion-weighted imaging was found to be helpful in detection of parenchymal laceration similar to post-contrast scans [31]. Advanced imaging technologies, such as accelerated parallel imaging and motion-resistant reconstruction, can further optimize post-contrast sequences [31–35]. Black blood and bright blood sequences are non-contrast techniques that can be used when vessel assessment is required [33].

Contrast-enhanced ultrasound has been found to be a reliable tool in the detection of solid organ injury in hemodynamically stable children with low- to moderate-energy abdominal trauma. It can detect active bleeding, pseudoaneurysms, and vascular injury that may not be apparent on conventional ultrasound. It also serves as a valuable tool for clarifying ambiguous findings on contrast-enhanced CT scans and in monitoring conservatively managed patients [36]. However, its use is limited by the limited availability and cost of the contrast media, difficult examination of deep regions, and low sensitivity in detecting urological injury [37].

The American Association for the Surgery of Trauma (AAST) first introduced the Organ Injury Scale (OIS) guidelines in 1989 to classify injuries of the liver, spleen, and kidneys, with a subsequent update in 1994. The primary motivation for refining trauma grading systems is to improve their accuracy in predicting mortality and enhancing patient management [38, 39]. Another significant revision was introduced in 2018, categorizing solid organ injuries based on criteria derived from imaging, surgical findings, and pathological analysis. The final grade is determined by the highest-ranking criterion among the three [40]. Furthermore, if multiple low-grade (I or II) injuries are present within the same organ, the overall classification is upgraded to grade III. This update also includes vascular injuries as pseudoaneurysms and arteriovenous fistulas. Segmental vascular injury and renal infarction have been added to renal injury grading. Moreover, the term “nonexpanding” was removed for subcapsular, perinephric, and splenic hematomas, as a single radiologic examination cannot definitively rule out expansion. Additionally, grade VI hepatic avulsion was eliminated due to its nonsurvivable nature, making grade V the highest classification for liver injuries, a standard now uniformly applied to all three solid organs. Finally, the use of Couinaud segments in describing parenchymal injury has been eliminated as well [38, 39]. Grading of different solid organs is summarized in Supplementary Material 1.

This review highlights the 2018 revision of AAST-OIS and the implications of the grading system on patient management with special emphasis on pediatric trauma patients. The technique of CT and concerns about radiation exposure are discussed with illustrations of different injury grades. This is to act as a comprehensive guide for radiology residents in the emergency setting. This grading system ranges from grade I, representing minor injuries, to grade V, which includes extensive lacerations, major vascular injuries, or avulsion [40, 41].

## Implications of the grading system on patient management

Both the American Pediatric Surgical Association and the Western Trauma Association acknowledge the value of CT in the assessment and grading of abdominal trauma by using AAST-OIS [42–44]. Both guidelines recognize that CT injury grading and vascular injury correlate with non-operative management failure, but their impact on treatment decisions is more significant in adults. In pediatric patients, management is determined solely by hemodynamic stability, as stable children continue with non-operative management and are admitted to the ward regardless of CT findings. AAST grades may be more related to decision management in terms of hospital stay and restrictions on physical activity [4, 40, 44]. By adopting the AAST-OIS grading system, healthcare professionals can communicate through a standardized framework, fostering better interprofessional collaboration and improving patient outcomes.

For adults, guidelines recommend observation for stable patients with low-grade injuries while advising that those with vascular injuries should undergo angioembolization. Patients with grade III-V parenchymal injuries without vascular involvement may also benefit from angioembolization. In cases of splenic trauma, the reported surgical intervention rate is less than 6% in children, whereas it reaches 20% in adults. While angioembolization is a standard procedure for adults, it is seldom employed in pediatric splenic trauma [45–47].

## Computed tomography imaging technique

Contrast-enhanced CT is the standard of care imaging modality for the assessment of visceral and solid organ injury. Initially, monitoring devices and ECG leads should be removed prior to scanning to avoid streak artifacts. Owing to the rapid scanning time of CT, sedation is usually not indicated. Additionally, skilled child life specialists minimize the fear and anxiety of injured children, obviating the need for sedation [4]. Distraction tools can enhance cooperation and reduce anxiety in pediatric patients. Utilizing projectors with child-friendly themes, playing music, providing toys with flashing lights or sounds, displaying engaging images on the ceiling or walls, singing, counting, and allowing parents to read or talk to the child through the console can all contribute to a more comforting experience [48].

To enhance imaging quality while minimizing CT radiation exposure, various strategies have been implemented [48]. Image parameters should be adjusted to suit pediatric patients, ensuring that the field of view and collimation are tailored to their smaller size. Proper positioning is essential, with the child centered in the gantry. Using a posteroanterior projection for the scout image in supine patients helps reduce radiation exposure to radiosensitive organs. Additionally, the product of tube current and exposure time (mAs), KVp, and pitch should be optimized based on the patient’s size with acceptable level of noise. Automatic exposure control allows the tube current to adjust according to the X-ray path length through the body, but its use can be complex and requires careful application [22, 48]. The field of view of the CT scans should be restricted to the necessary anatomical areas, typically covering the region from the lower thoracic cavity to the level of the symphysis pubis.

**Fig. 1:**
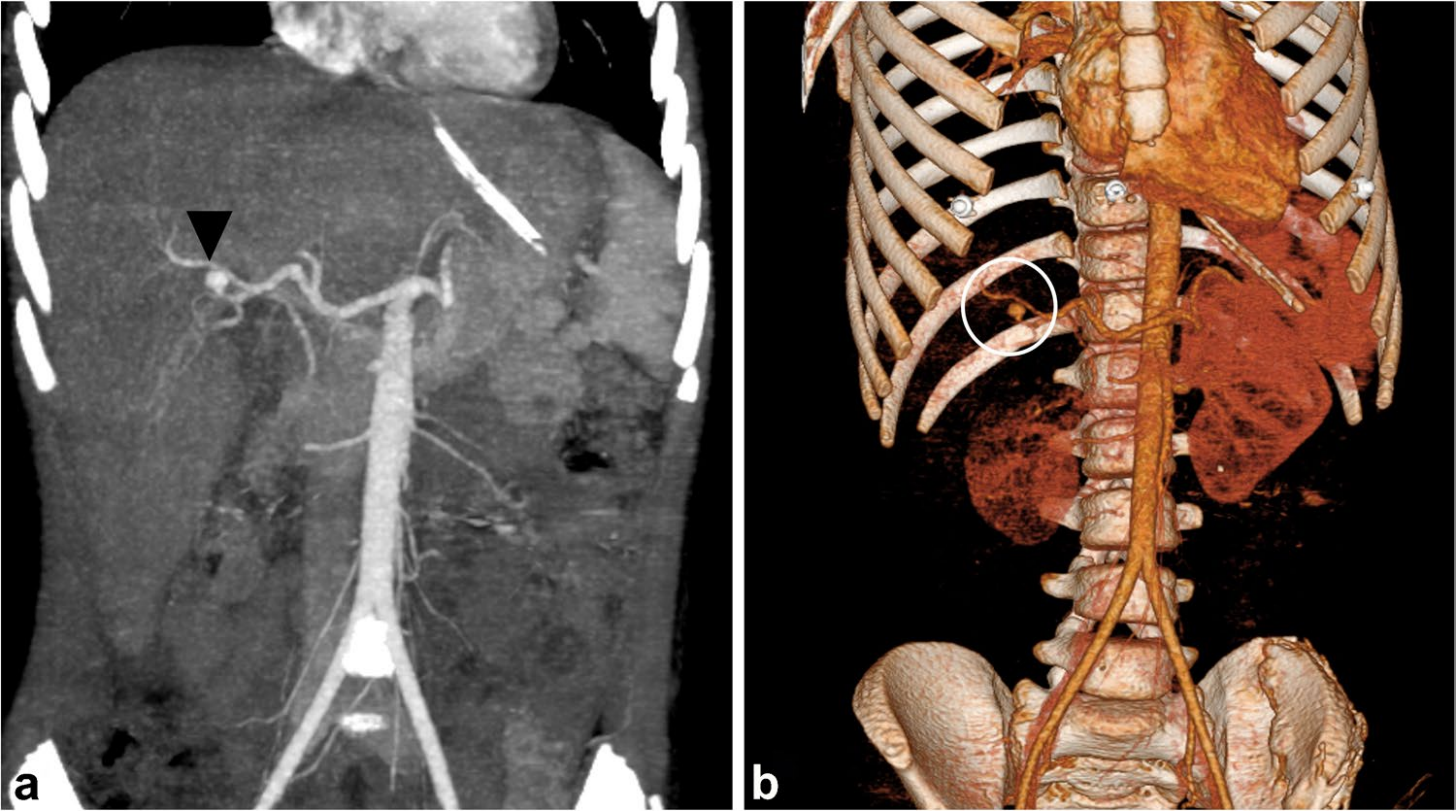
An 11-year-old boy complains of right hypochondrial pain. He gave a history of blunt abdominal trauma a month ago. Ultrasound and Doppler showed pseudoaneurysm (*not shown*). Coronal computed tomography maximum intensity projection at the arterial phase shows a small, well-defined hyperattenuating rounded focus (*arrowhead*) arising from the posterior branch of the right hepatic artery, which then fades in the delayed phase (*not shown*), indicating vascular injury (hepatic artery traumatic pseudoaneurysm) (grade III injury). **b** Three-dimensional reconstruction depicts a small hepatic pseudoaneurysm (*circle*) arising from a right hepatic artery branch

Concerns about radiation exposure, particularly in pediatric patients with blunt abdominal trauma, support the use of CT imaging in a single phase (most commonly the venous phase) [49]. Multiphase acquisitions contribute to unnecessarily high radiation doses. Unlike adult protocols [50, 51], routine arterial phase imaging is not standard practice in children [49–52]. To optimize contrast enhancement while minimizing radiation exposure, especially when vascular assessment is necessary—such as in cases of suspected active bleeding or vascular injury—a split-bolus technique has been developed [53, 54]. This method involves two sequential contrast boluses: the first, two-thirds contrast volume administered slowly, enhances solid organs and the portal venous system; the second, one-third volume injected rapidly, provides angiographic visualization of the arterial system. A single scan is then performed to capture both phases [54]. To assist in accurate dose and rate calculations, the Camp-Bastion contrast wheel and a user-friendly calculator have been introduced [54, 55]. Nonetheless, in selected clinical contexts—particularly high-velocity or penetrating abdominal trauma—an arterial phase scan may be justified to evaluate suspected arterial injuries and guide endovascular intervention (Fig. 1) [54, 56–58].

Delayed imaging might be necessary if initial scans reveal a vascular blush within a solid organ or retroperitoneal hematoma. To distinguish active bleeding from pseudoaneurysms and arteriovenous fistulae, delayed imaging after a short interval (5–10 min) may be helpful. Hematomas with active extravasation typically show persistent or increasing contrast accumulation, while pseudoaneurysms and arteriovenous fistulae tend to exhibit contrast washout or dilution [51, 58, 59].

If injury to the urinary system is suspected, a delayed scan may be added. To further reduce radiation exposure, a modified version of the split-bolus technique can be employed. In this method, the contrast bolus is divided into three separate portions and administered at staggered intervals, commonly at 10 min, 90 s, and 30 s prior to imaging. This strategy enables the simultaneous assessment of multiple types of injuries in a single acquisition, thereby minimizing the overall exposure to ionizing radiation [60]. However, urinary bladder injuries may go undetected when the bladder is inadequately filled, in which case a CT cystogram may be necessary. Injury of the urinary system is clinically suspected when there is macroscopic hematuria, pelvic fractures, or associated renal lacerations grade II or higher, or when there is fluid collection around the renal pelvis. Delayed images should not be ordered routinely to limit radiation exposure and to shorten the examination time in critically ill patients [51].

It was postulated that diluted (2%) oral water-soluble contrast media may be used for better detection of intramural hematoma, mesenteric injury, and bowel perforation by detection of contrast extravasation. However, its use is limited due to decreased bowel motility in children with abdominal trauma, which increases the risk of vomiting and aspiration. The air‒contrast interface may induce artifacts, which may degrade the imaging quality. Additionally, the use of oral contrast media does not increase the diagnostic accuracy of CT in the context of abdominal trauma [4, 61].

Dual-energy CT is an emerging imaging technique with the advantage of lower radiation dose, higher temporal resolution, and good-quality images [62]. Low-peak-voltage datasets enhance iodine contrast especially in pediatric patients who have minimal visceral fat and assist in distinguishing congenital cleft versus laceration. On the other hand, higher peak-voltage settings are beneficial in minimizing beam hardening and metal-related artifacts. Virtual non-contrast imaging can be used to identify and localize blood products, enabling precise injury grading. An added advantage of using dual-energy CT is its ability to distinguish between pseudoaneurysm/arteriovenous fistula versus active bleeding [63].

## Imaging nomenclature in the setting of trauma

To improve communication between radiologists and other medical professionals, a common language for describing radiological findings in trauma patients is necessary. It increases the accuracy of diagnoses and grading of trauma. Definitions of lacerations, hematoma and contusion, active bleeding, pseudoaneurysm, fracture of a solid organ, and sentinel clot are summarized in Table 2 [4, 51, 64–66]. The grading of solid organ injury is summarized in Supplementary Material 2.

**Table 2:** Important lexicon in trauma imaging

Term	Definition
Laceration	Linear or branching hypodense area
Fracture	Laceration that extends from one capsular surface to the other
Subcapsular hematoma	Crescentic hypodense area that usually compresses the nearby parenchyma
Parenchymal hematoma	Intraparenchymal focal area of hypoattenuation
Contusion	Ill-defined hypoattenuating area during the portal venous phase that becomes isoattenuating in the delayed phase
Devascularization/infarction	Wedge-shaped or diffuse area of hypoattenuation that fails to show contrast enhancement in all phases
Sentinel clot	A relatively hyperattenuating fluid adjacent to the site of solid organ or visceral injury
Active bleeding	Focal or diffuse contrast blush (> 90 HU) that increases in size or density in the delayed phase. It does not follow the blood pool Arterial bleeding: the attenuation of extravasated blood is similar to or more than the attenuation in the arterial phase In venous bleeding: the attenuation is more appreciated in the delayed phases than in the arterial phase
Contained vascular injury (pseudoaneurysm and arteriovenous fistula)	Sharply defined focal collection of contrast that decreases in density in the delayed phase. It follows the attenuation of the blood pool Simultaneous enhancement of the artery and nearby vein, with asymmetric early filling of the affected vein compared to the unaffected vein, is suggestive of arteriovenous fistula

References [4, 51, 64–66]

## Practical tips for imaging interpretation

**Fig. 2:**
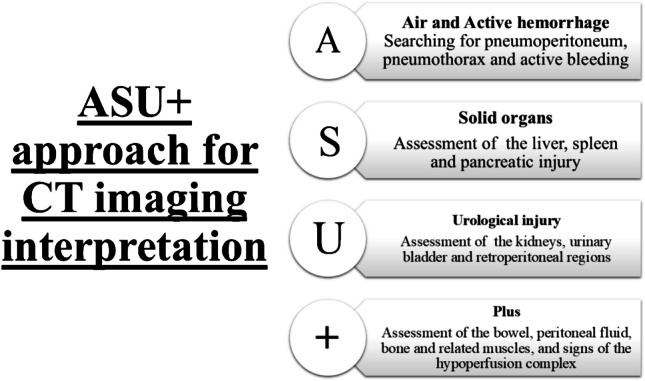
The extended air and active hemorrhage, solid organ injury, and urological injury (ASU +) approach for interpretation of abdominal trauma computed tomography scans

Similar to the step-by-step approach for CT scan interpretation described by San Francisco General Hospital [63], a simplified approach (ASU ^+^) has been developed in our hospital for the assessment of abdominal trauma scans (Fig. 2): A: (Air and Active hemorrhage) searching for pneumoperitoneum, pneumothorax, and active bleeding; S: (Solid organ) for the assessment of liver, spleen, and pancreatic injury; U: (Urological injury) for the assessment of the kidney, urinary bladder, and retroperitoneal regions, including the adrenal glands, aorta, and IVC and their branches; + : (plus) for the assessment of the bowel, peritoneal fluid, bone and related muscles, and signs of the hypoperfusion complex.

Using appropriate window levels and multiplanar reformat is crucial for proper assessment and for accurate depiction of all radiological signs [64]. The lung window is adopted for the detection of air (A) in the pleura and peritoneum (pneumothorax and pneumoperitoneum). Intra-abdominal air may be observed in the retroperitoneum or inside the peritoneal cavity. Searching for air adjacent to the anterior surface of the liver facilitates the detection of free air [61]. Air may also be found adjacent to the bowel loops. The liver window is used for the assessment of the liver parenchyma. The soft tissue window is used for the detection of active hemorrhage (A), soft tissue or solid organ injury (S), and the search for peritoneal fluid. Active hemorrhage appears as a high-attenuation (isodense to enhanced arteries) blush that persists into delayed images and does not follow the blood pool [66]. Due to tamponade, thrombosis, or spasm, injured vessels may bleed intermittently, potentially leading to false-negative CT results. Therefore, careful interpretation of abdominal CT scans is essential, especially when a large hematoma is present. A sentinel clot (high-density hematoma more than 60 HU) indicates injury to a nearby organ [67]. Peritoneal fluid can indicate either solid organ or bowel injury. Small amounts of hemoperitoneum in the hepatorenal space, paracolic gutters, or pelvis may be overlooked [66]. A thorough assessment of these areas is essential for accurate detection [64]. Furthermore, a common challenge in assessing abdominal trauma is that beam hardening artifacts from structures like ribs or nasogastric tubes may create false positives for contusions or lacerations in nearby organs [64]. Another potential pitfall is that gonadal veins can sometimes be mistaken for ureters. To avoid this error, their anatomical course should be carefully followed, and delayed phase imaging should be performed [66, 68]. Finally, the bone window is used to search for fractures of the ribs, vertebrae, pelvic bones, or visualized parts of the femur bones.

Multiplanar reformat in coronal and axial planes plays a complementary role in the interpretation of different imaging findings, particularly those related to vascular and diaphragmatic injury [66]. Maximum intensity projection (MIP) is commonly used to visualize high-attenuation data voxels. MIP is implemented with PACS viewing system without the need for dedicated workstations. Three-dimensional reconstruction is helpful in the evaluation of vascular anatomy and related injuries by providing virtual views for interventional approaches [66].

Signs of hypovolemic shock (hypoperfusion complex) include diminished calibres of the aorta and IVC; diffuse intense enhancement of the bowel wall, mesentery, pancreas, and suprarenal glands; peritoneal and retroperitoneal fluid collection with dilated and thick wall bowel loops; and periportal low attenuation [4].

The reporting template includes the patient’s clinical history, imaging technique, radiological signs, and description of the solid organ injury according to the AAST-OIS and is then finalized by clear determination of whether the study is normal or abnormal, with accurate grading of the identified injury [18, 54].

## Future directions

Artificial intelligence techniques have shown a promising role in enhancing radiologists’ diagnostic efficiency and accuracy, which may improve patient outcomes [69]. However, few studies in the literature have investigated the validity of machine learning models in interpreting trauma CT scans. Eight RSNA 2023 Abdominal Trauma AI Challenge award-winning models demonstrated excellent performance in identifying traumatic abdominal injuries, especially high-grade injuries. These technologies may be particularly helpful for triaging patients, providing a second opinion, and identifying locations with limited resources and the unavailability of radiologists [70].

## Liver trauma

**Fig. 3:**
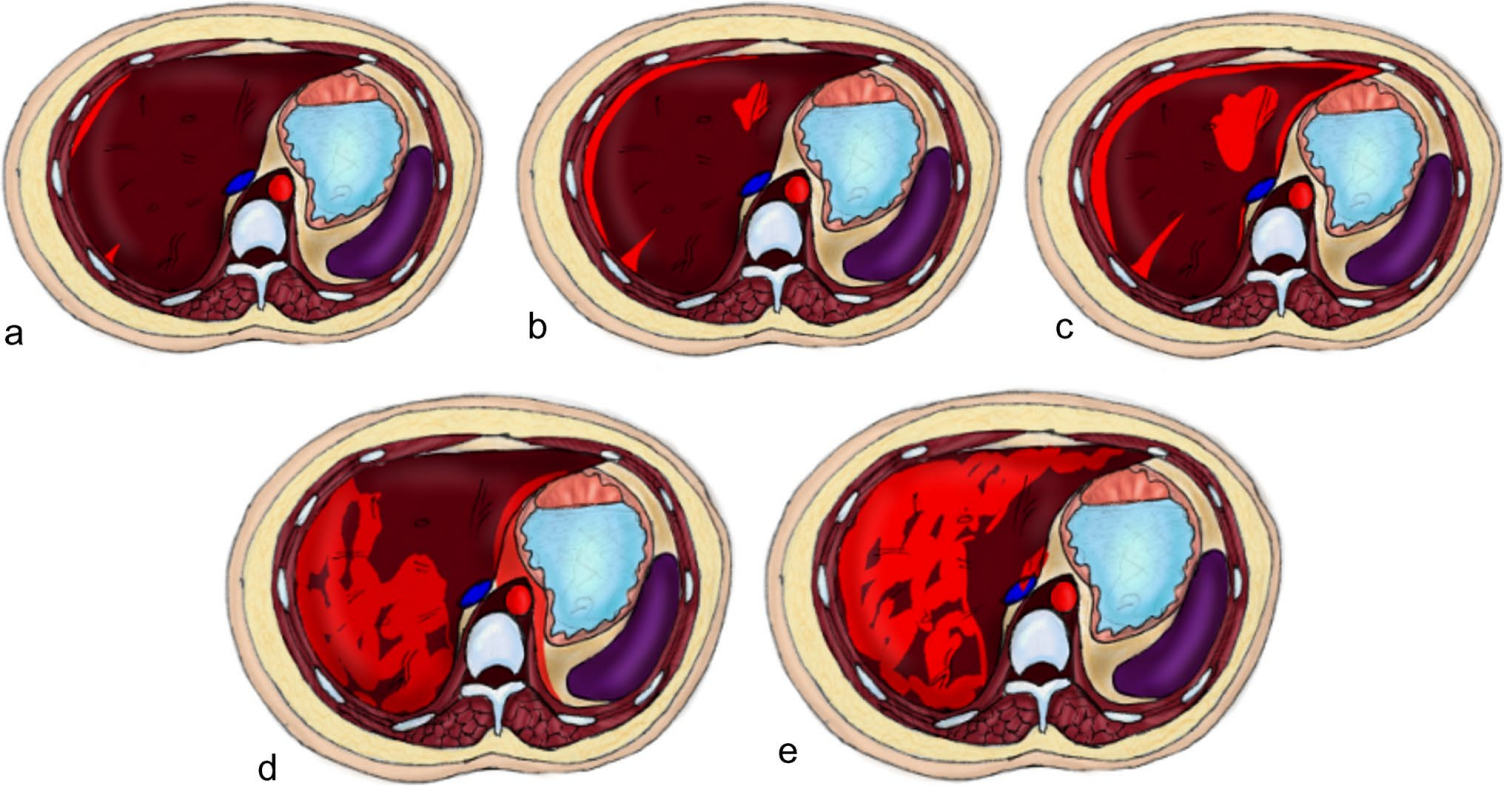
A diagram depicting the five grades of liver injury grading system. **a** Grade I injuries with either a subcapsular hematoma of less than 10% of the hepatic surface area or parenchymal lacerations of less than 1 cm depth. **b** Grade II injuries include subcapsular hematomas involving 10–50% of the hepatic surface area, parenchymal hematomas less than 10 cm in diameter, or lacerations 1–3 cm in depth and less than or equal to 10 cm in length. **c** Grade III injuries with subcapsular hematoma involving more than 50% of the hepatic surface area, parenchymal hematoma with a diameter greater than 10 cm, ruptured subcapsular or parenchymal hematoma, lacerations greater than 3 cm in depth, and vascular injuries containing active bleeding. **d** Grade IV injuries include injuries causing parenchymal disruption of 25–75% of a hepatic lobe or vascular injuries with active bleeding extending into the peritoneum. **e** Grade V injuries include hepatic lobe parenchymal disruption of greater than 75% and/or injuries to the retrohepatic vena cava or major hepatic veins

**Fig. 4:**
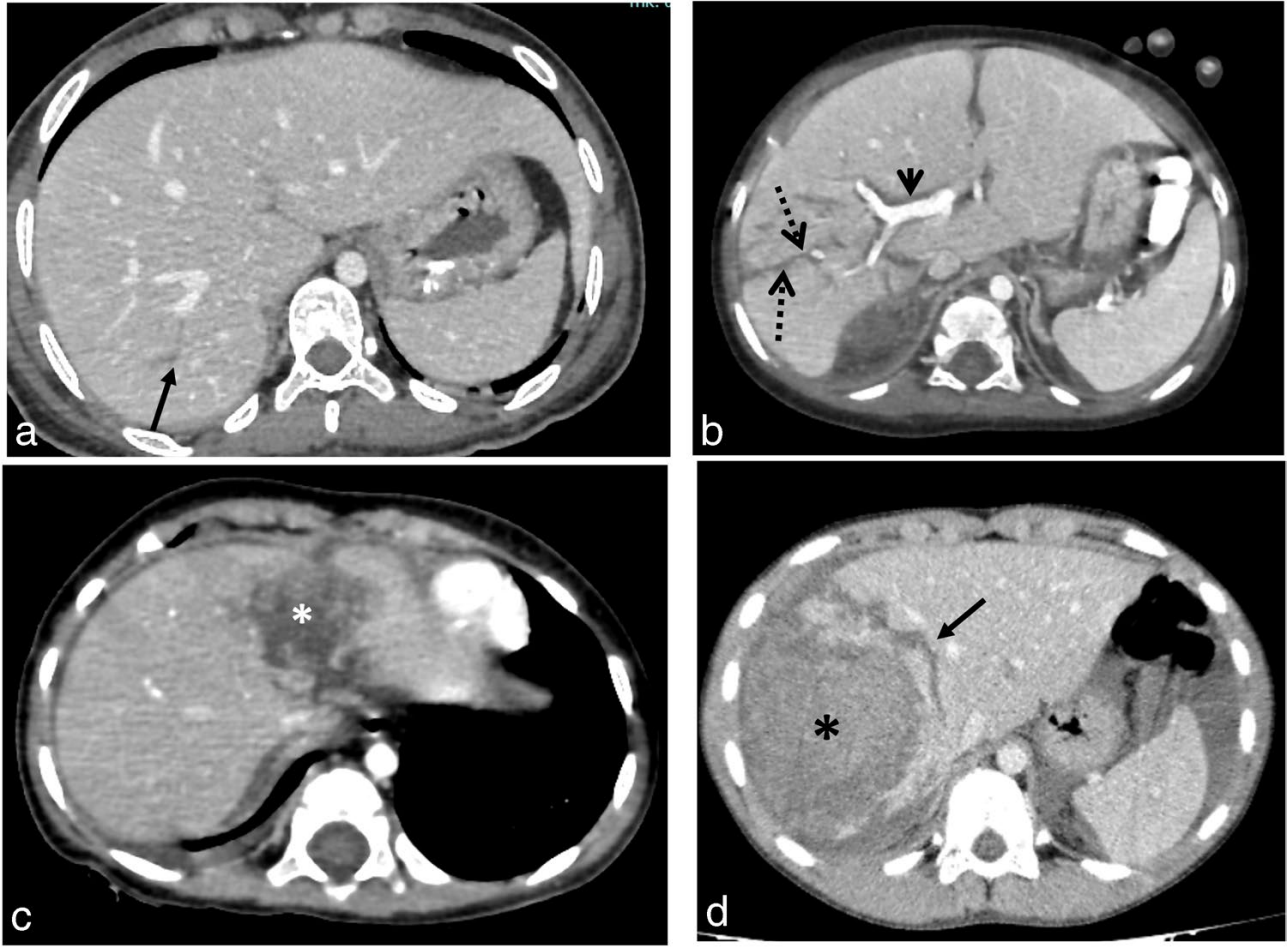
Axial computed tomography at the portal venous phase of four different patients having different grades of liver injury. **a** An 18-year-old girl with grade II injury with a thin linear capsular laceration of 1–3 cm parenchymal depth (*black arrow*). **b** A 2-year-old boy with grade III hepatic injury showing a deep branching intraparenchymal laceration greater than 3 cm deep (*broken arrows*). Note the periportal hypoattenuation (*short arrow*). **c** A 2-year-old boy with hepatic intraparenchymal hematoma less than 10 cm in diameter (*asterisk*) represents a grade III injury. **d** An 11-year-old boy with a larger intraparenchymal hematoma more than 10 cm in diameter (*asterisk*) with disruption of more than 75% of the liver parenchyma of the right lobe, representing grade V hepatic injury. Note the nearby deep laceration (*arrow*)

In the context of abdominal trauma, the liver is the most frequently injured organ in many series or the second most frequently injured organ in others. It is associated with splenic injuries in approximately one-third of patients [4, 51, 64]. Liver trauma grades range from grade 1 where there is minor laceration or subcapsular hematoma to grade V where there is major disruption (75%) of the hepatic parenchyma or IVC or major hepatic veins (Figs. 3 and 4) (Supplementary Table 1). Compared with free peritoneal perihepatic hematoma, subcapsular hematoma results in mass effect on the underlying liver parenchyma [64]. The posterior segment of the right lobe is the most frequently injured area of the liver because it is fixed with the coronary ligaments. Injury to the bare area of the liver may lead to retroperitoneal hematoma. Parenchymal hematoma extends to the peritoneum when associated with capsular tear in two-thirds of cases [4]. The periportal zone of hypoattenuation may be visualized in children with abdominal trauma (Fig. 3). This zone represents dilated periportal lymphatics due to extensive fluid resuscitation. Periportal tracking of blood may appear as hypoattenuating linear zones adjacent to the enhanced portal vein [64].

**Fig. 5:**
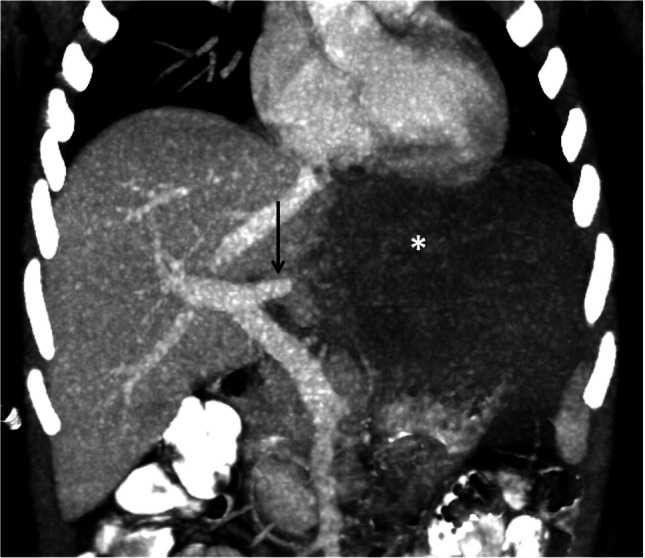
A 5-year-old boy with complete devascularization of the left lobe of the liver. Coronal maximum intensity projection at the portal venous phase showing nonenhancement of the left lobe of the liver (*asterisk*) with abrupt termination of the left branch of the portal vein (*arrow*) (grade V injury)

**Fig. 6:**
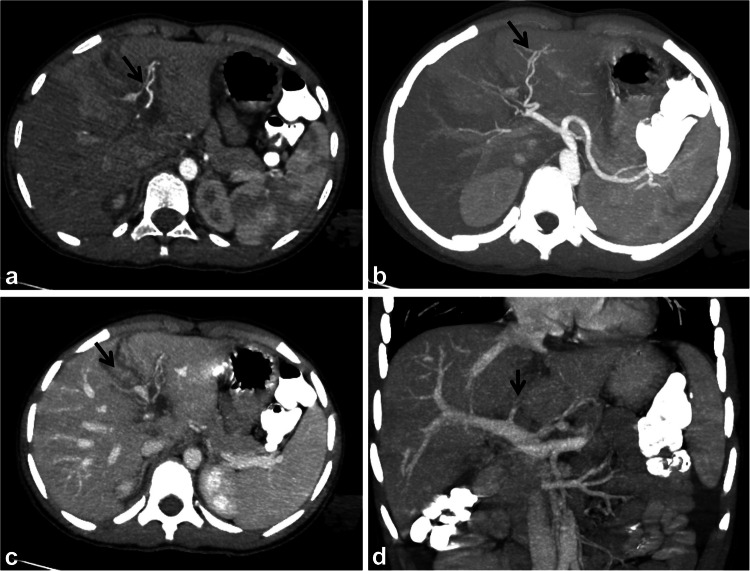
A 9-year-old boy with an arteriovenous fistula following a road traffic accident (grade III injury). **a**, **b** Axial section (**a**) and maximum intensity projection (*MIP*) (**b**) during the arterial phase of the computed tomography scan show simultaneous enhancement of the hepatic artery and nearby medial branch of the left portal vein (*arrow*). Note the abnormal early enhancement of the left lobe of the liver. **c** Axial section at the portal venous phase shows multiple deep lacerations (*arrow*) extending to the porta hepatis. **d** Coronal MIP reconstruction showing opacification of an attenuated short stump of the left portal vein (*arrow*)

Although rare in children, hepatic vascular injury may occur. Hepatic devascularization may occur due to injury to the arterial and portal venous blood supply. Arteriovenous fistulas and pseudoaneurysm may also occur (Figs. 1, 5, and 6) [17].

**Fig. 7:**
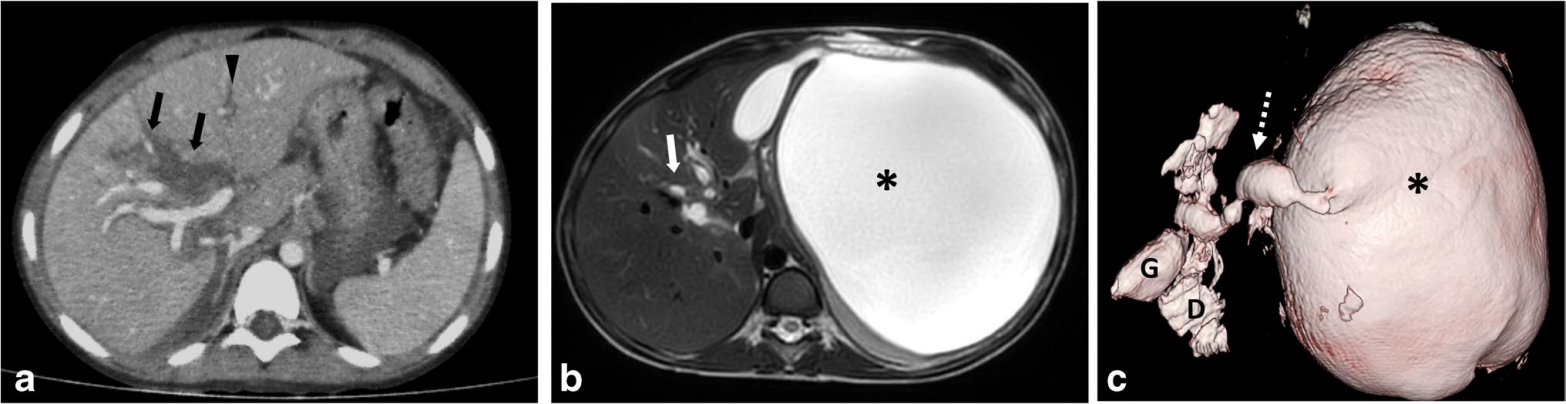
A 6-year-old boy with abdominal trauma. **a** Axial contrast-enhanced computed tomography at the time of injury shows a deep laceration (*arrow*) in the right hepatic lobe (grade III injury) reaching the hilar region with intrahepatic biliary radicle dilatation (*arrowhead*). **b**, **c** A repeat magnetic resonance imaging after 1 month axial T2-weighted image (**b**) and three-dimensional reconstruction of the magnetic resonance cholangiopancreatography (**c**) show a large left hypochondrial cystic lesion (*asterisk*). It is connected to dilated biliary radicles (*arrow*) by a small track (*broken arrow*), indicating traumatic biloma formation. *D*, duodenum; *G*, gallbladder

Gallbladder and extrahepatic biliary injuries are relatively rare (reported incidences ranging from 0.9% to 3.7% of pediatric blunt hepatic injuries). It ranges from gallbladder hematoma (grade I) to more than 50% transection of the common bile duct or injury to the intraduodenal or intrapancreatic bile duct (grade V) [40, 51]. Owing to the conservative management of most hepatic injuries, most biliary injuries are discovered late. On CT, the presence of parenchymal injury extending to the portal hepatis (Fig. 7), which is associated with contracted gall bladder in a fasting patient, is highly suggestive of biliary injury. Additionally, the presence of a thick or ill-defined gallbladder wall and dense intracystic fluid is suggestive of gall bladder injury [40, 71]. The diagnosis may be delayed up to 9 days or 60 days, and MRI may be valuable in this instance (Fig. 7). MRI allows for accurate localization and assessment of the extent of biliary injury. This can be visualized not only through magnetic resonance cholangiopancreatography (MRCP) sequences but also after administering hepatobiliary-specific contrast agents. Detection of extraluminal contrast around the liver or in the peritoneal cavity is diagnostic of biliary system injury. The hepatobiliary phase is typically obtained 90 min to 120 min following intravenous injection of gadobenate dimeglumine or 15 min to 20 min after administering gadoxetic acid [31].

## Spleen trauma

Splenic injury often results from blunt abdominal trauma, and many cases are successfully managed nonoperatively, particularly in hemodynamically stable children [44]. Close monitoring and supportive care, including transfusions, if necessary, are crucial to avoid unnecessary surgeries. However, in cases of significant hemorrhage or complications, surgical intervention, such as splenectomy or splenic preservation techniques, may be needed [4, 40, 44].

Owing to its small size, severe trauma may lead to splenic shuttering. Hemoperitoneum may be associated with splenic injury if there is capsular tear. Blood may extend to the retroperitoneum if the splenic hilum is injured. Blood tracks along the splenorenal ligament to the anterior pararenal space [4].

**Fig. 8:**
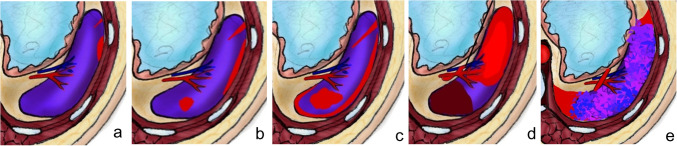
Diagram illustrating splenic trauma grading system.** a** Grade I splenic injury, which includes a small subcapsular hematoma involving less than 10% of the splenic surface area or any capsular tear of less than 1 cm depth. **b** Grade II injury with a subcapsular hematoma involving approximately 10–50% of the surface area. It also involves small intraparenchymal hematomas less than 5 cm in diameter and capsular tears spanning approximately 1–3 cm in depth. **c** Grade III splenic injuries, including larger subcapsular hematomas occupying more than 50% of the surface area, ruptured subcapsular or parenchymal hematomas, parenchymal hematomas greater than or equal to 5 cm in diameter, or capsular lacerations greater than 3 cm in depth. **d** Grade IV involves more aggressive injuries, such as tears involving segmental or hilar vessels causing more than 25% splenic devascularization; vascular injuries, including arteriovenous fistulas or pseudoaneurysms; and confined active bleeding inside the capsule. **e** Grade V includes a shattered spleen and vascular injuries causing active bleeding that extends into the peritoneum

**Fig. 9:**
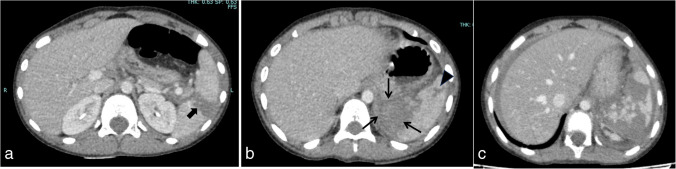
Axial contrast-enhanced computed tomography of three different children with abdominal trauma. **a** A 9-year-old boy with a laceration (*arrow*) extending from one capsular surface to the other (fracture) for more than 3 cm denoting grade III injury. **b** An 8-year-old boy with parenchymal hematoma (*arrows*) associated with a short laceration (*arrowhead*), with no evidence of contrast extravasation or major vascular injury, denoting grade III splenic injury. **c** Multiple lacerations and parenchymal hematoma in a 9-year-old boy with a shattered spleen denoting grade V injury

**Fig. 10:**
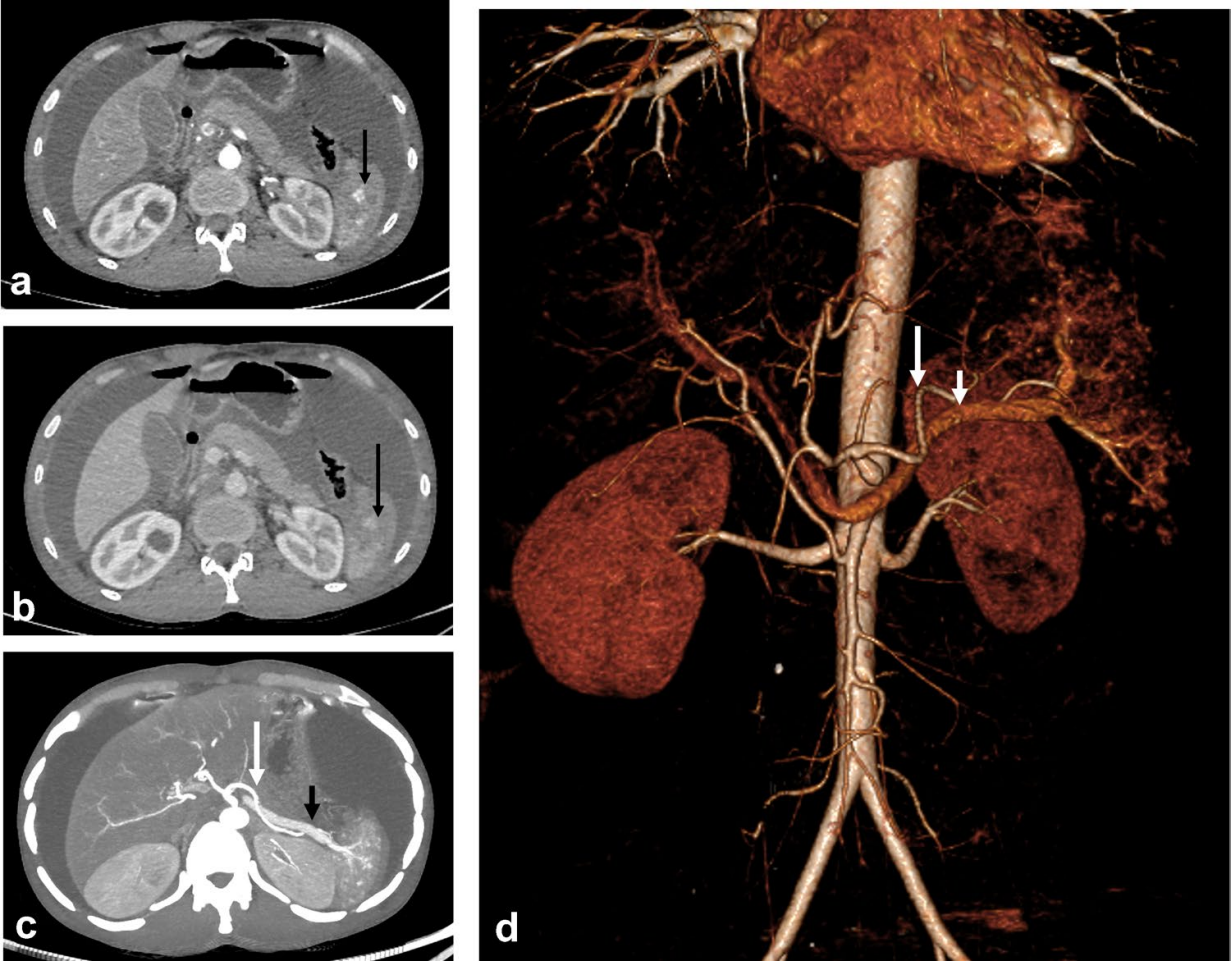
An 18-year-old boy with a road traffic accident with post-traumatic arteriovenous fistula. **a**, **b** Axial contrast-enhanced computed tomography at the arterial (**a**) and venous phases (**b**) shows contained vascular injury (*arrow*) in the spleen following the arterial pool. **c**, **d** Axial maximum intensity projection (**c**) and three-dimensional reconstruction (**d**) show simultaneous enhancement of the splenic vein (*short arrow*) with the splenic artery (*long arrow*) indicating the development of an arteriovenous fistula (grade IV injury)

Splenic trauma is categorized into five grades based on the severity of the injury (Fig. 8). Grade I represents minor injuries, such as superficial lacerations and contusions, and grade V indicates the most severe injuries, including complete splenic rupture and extensive vascular injuries (Figs. 9 and 10) (Supplementary Table 1) [40]. Splenic clefts are normal anatomical variants with a smooth linear low-density appearance and may be mistaken for lacerations. Lacerations usually have irregular outlines and are surrounded by hematomas, whereas larger clefts may contain fat [4].

## Urological trauma

Owing to their relatively larger size and position in the abdomen, kidneys are particularly vulnerable to injury in children. The kidneys are the third most frequently injured organ in children. Symptoms of renal trauma may vary depending on the severity of the injury but often include flank pain, hematuria, swelling or bruising, nausea, vomiting, hypotension, and tachycardia [4].

**Fig. 11:**
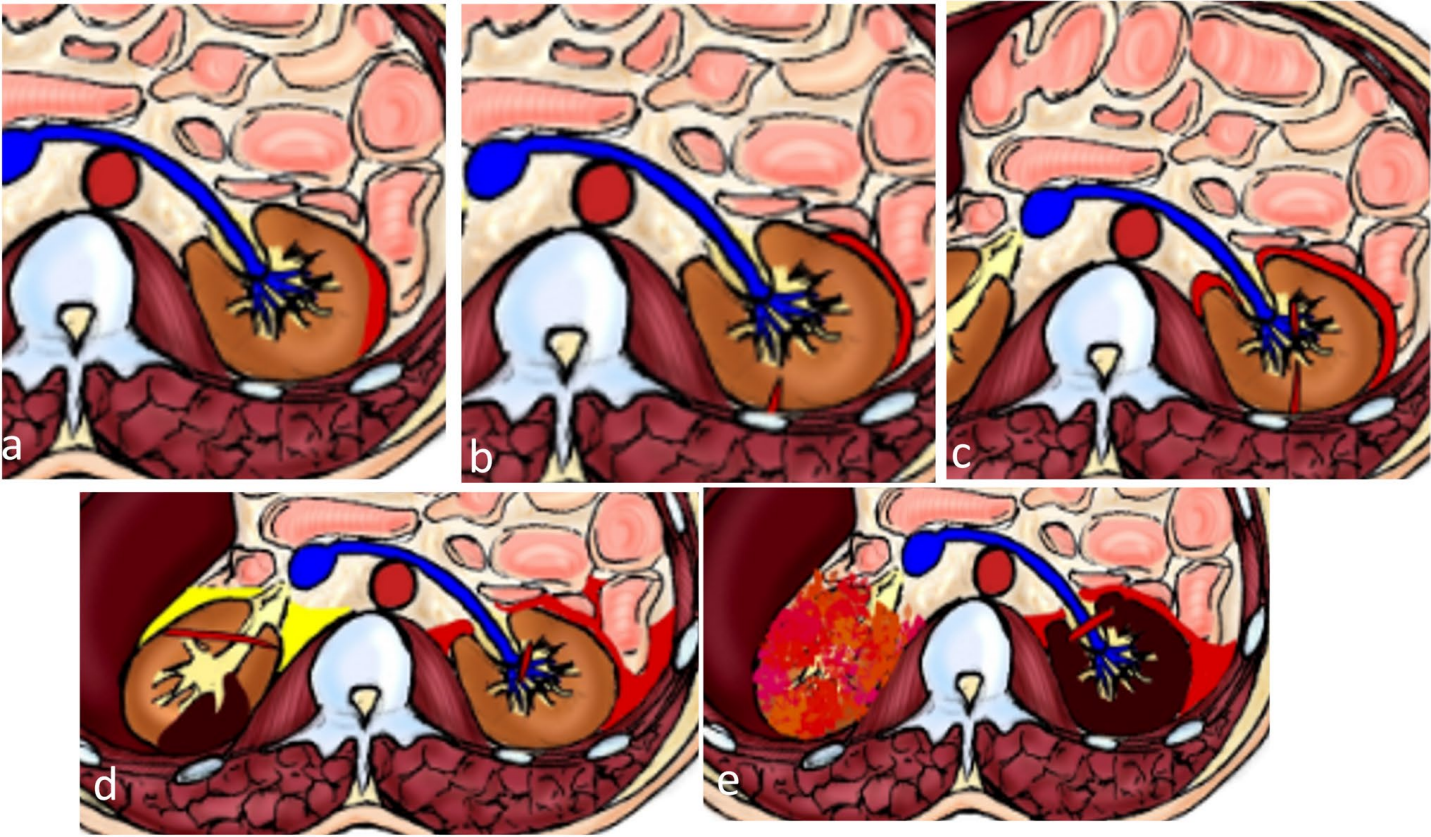
Diagram illustrating renal trauma grading. **a** Grade I injuries representing subcapsular hematomas (without laceration) and parenchymal contusions. **b** Grade II injuries, including perinephric hematoma confined to the Gerota fascia or lacerations equal to or less than 1 cm deep. **c** Grade III renal injuries include deep renal parenchymal lacerations more than 1 cm deep without rupturing of the collecting system or urine leakage. Grade III injuries include vascular injury or active bleeding within the Gerota fascia. **d** Grade IV injuries involve renal laceration with urine leakage due to extension into the urinary collecting system, renal pelvis laceration, or complete ureteropelvic junction disruption. It also includes segmental renal arterial or venous injury, active bleeding extending beyond the Gerota fascia, and segmental renal infarction because of vascular thrombosis (not active bleeding). **e** Grade V injuries, including a shattered kidney with disrupted anatomical delineation, an avulsed hilum, or laceration of the main renal artery or vein, and active bleeding with complete renal devascularization

**Fig. 12:**
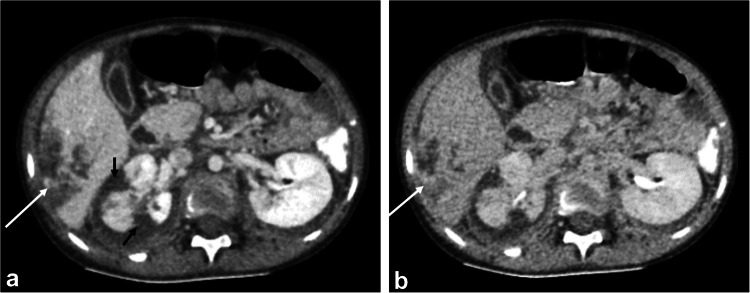
A 7-year-old girl with liver and renal injury following a motor traffic accident. **a**, **b** Axial contrast-enhanced computed tomography at the venous (**a**) and delayed (**b**) phases shows contained vascular injury (*white arrows*) following the blood pool (grade III injury). The surrounding hypoattenuating area becomes partially isoattenuating in the delayed phase, indicating liver contusion. Note the associated multiple wedge-shaped hypoattenuating areas (*black arrows*) in the right kidney that fail to show contrast enhancement in all phases denoting segmental infarctions (grade IV injury)

**Fig. 13:**
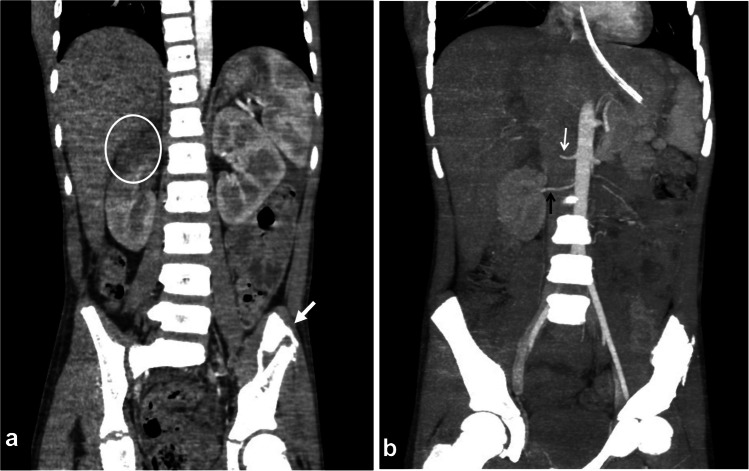
An 11-year-old boy with a history of road traffic accident. **a** Coronal contrast-enhanced computed tomography shows a hypodense area in the upper pole of the right kidney representing infarction (*circle*). Note the associated fracture of the left iliac bone (*arrow*)*.* **b** A coronal reconstructed maximum intensity projection reveals right renal artery transaction (*white arrow*) with proximal segment arterial enhancement followed by abrupt complete contrast cessation of the middle and distal arterial segments (grade V injury). The rest of the renal parenchyma shows normal enhancement due to the presence of an aberrant right renal artery (*black arrow*) arising from the aorta at the level of L2, supplying the middle and lower renal poles

**Fig. 14:**
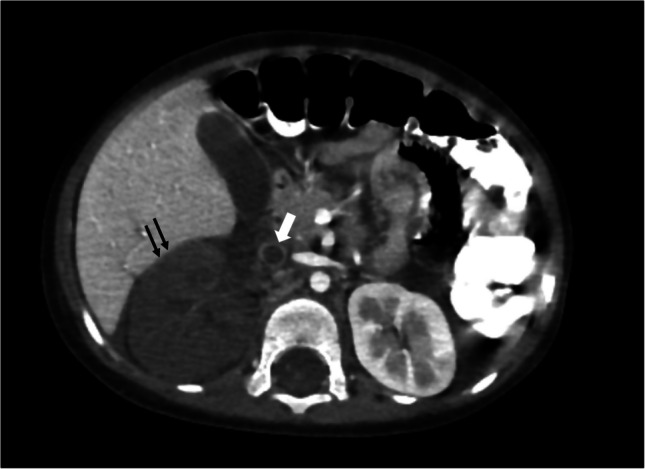
A 2-year-old boy with grade V renal injury. Axial contrast-enhanced computed tomography at the venous phase showing an enlarged right kidney (*black arrows*) with complete devascularization and non-enhancement of its parenchyma. The right renal artery was patent (*not shown*). The renal vein is distended, with an intraluminal filling defect (*white arrow*) extending to its draining segment at the inferior vena cava. There is associated mild perihepatic and perinephric fluid, with no signs of active extravasation

Renal injuries are classified into grades I to V, with lower grades typically involving minor contusions or lacerations and higher grades encompassing more severe injuries such as renal pedicle vascular injury or complete laceration (Fig. 11) (Supplementary Table 1) [40]. Lacerations are of critical concern in renal injuries, as the presence of a short < 1-cm laceration is considered a grade II injury, while longer lacerations that extend to the renal pelvis increase the grade of injury. Urinary extravasation is usually limited to the pararenal space. Owing to the continuity of the perirenal space and perivesical space in some individuals, extravasated urine or perinephric hematoma may extend to the pelvis [72, 73]. Non-urographic MRI can still assess the severity of renal or ureteral trauma. When performed, excretory-phase post-contrast MRI can reveal pelvicalyceal and ureteral injuries [33]. Lacerations and urinary extravasation are usually managed conservatively, but surgical repair may be required if urinary obstruction ensues. Segmental devascularization and infarction are usually treated conservatively, but injury to the main renal artery requires prompt intervention to preserve renal function (Figs. 12, 13, 14) [72].

**Fig. 15:**
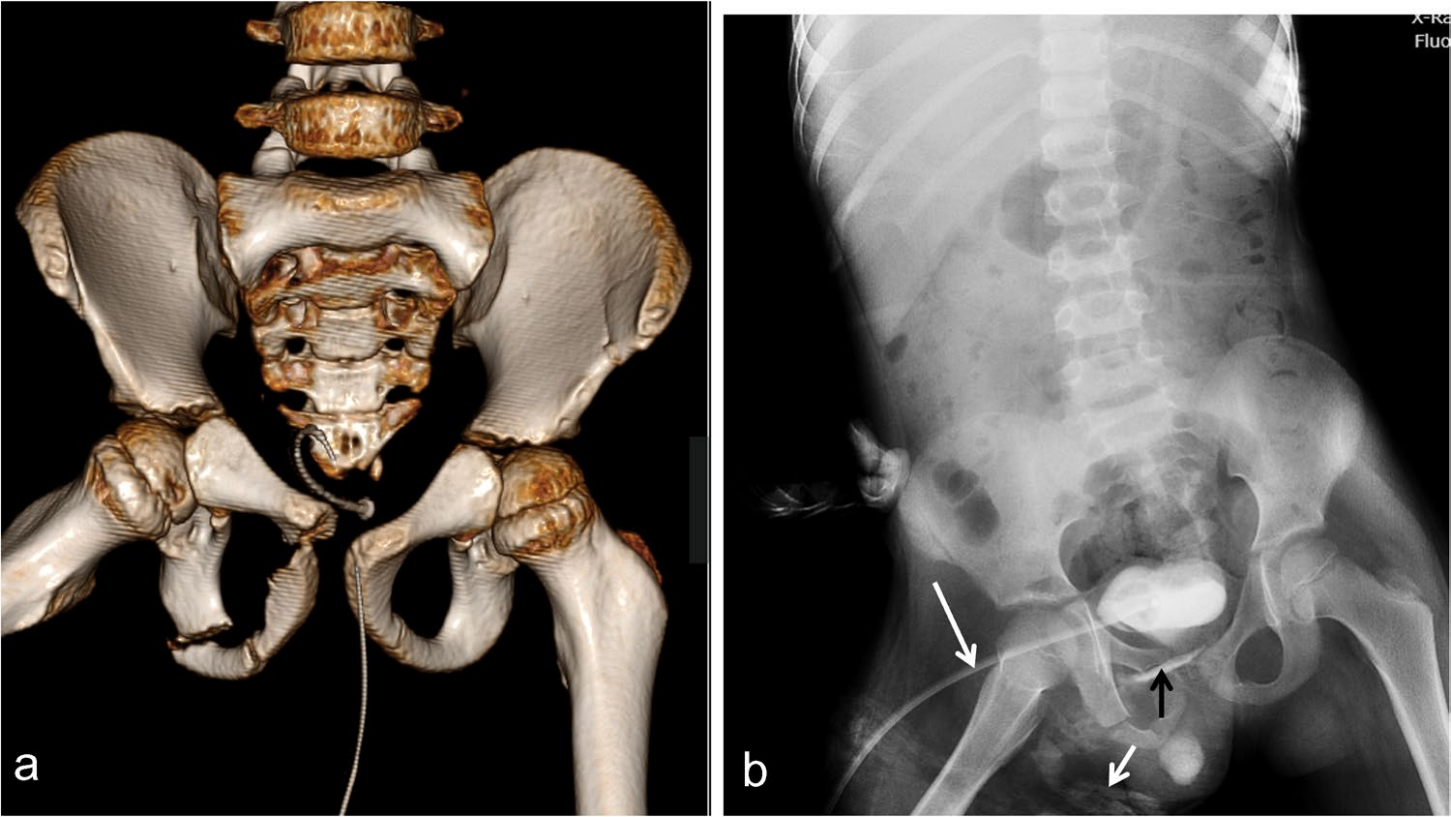
An 8-year-old boy with a fractured pelvis. **a** Computed tomography of the pelvis with three-dimensional reconstruction shows multiple fractures involving the right superior and inferior pubic rami associated with widening of the symphysis pubis. **b** Voiding cystourethrogram via suprapubic cystostomy (*long white arrow*) shows early contrast leakage at the lower aspect of the urinary bladder (*black arrow*) and the soft tissue of the perineum (*short white arrow*) indicating extraperitoneal rupture of the urinary bladder

**Fig. 16:**
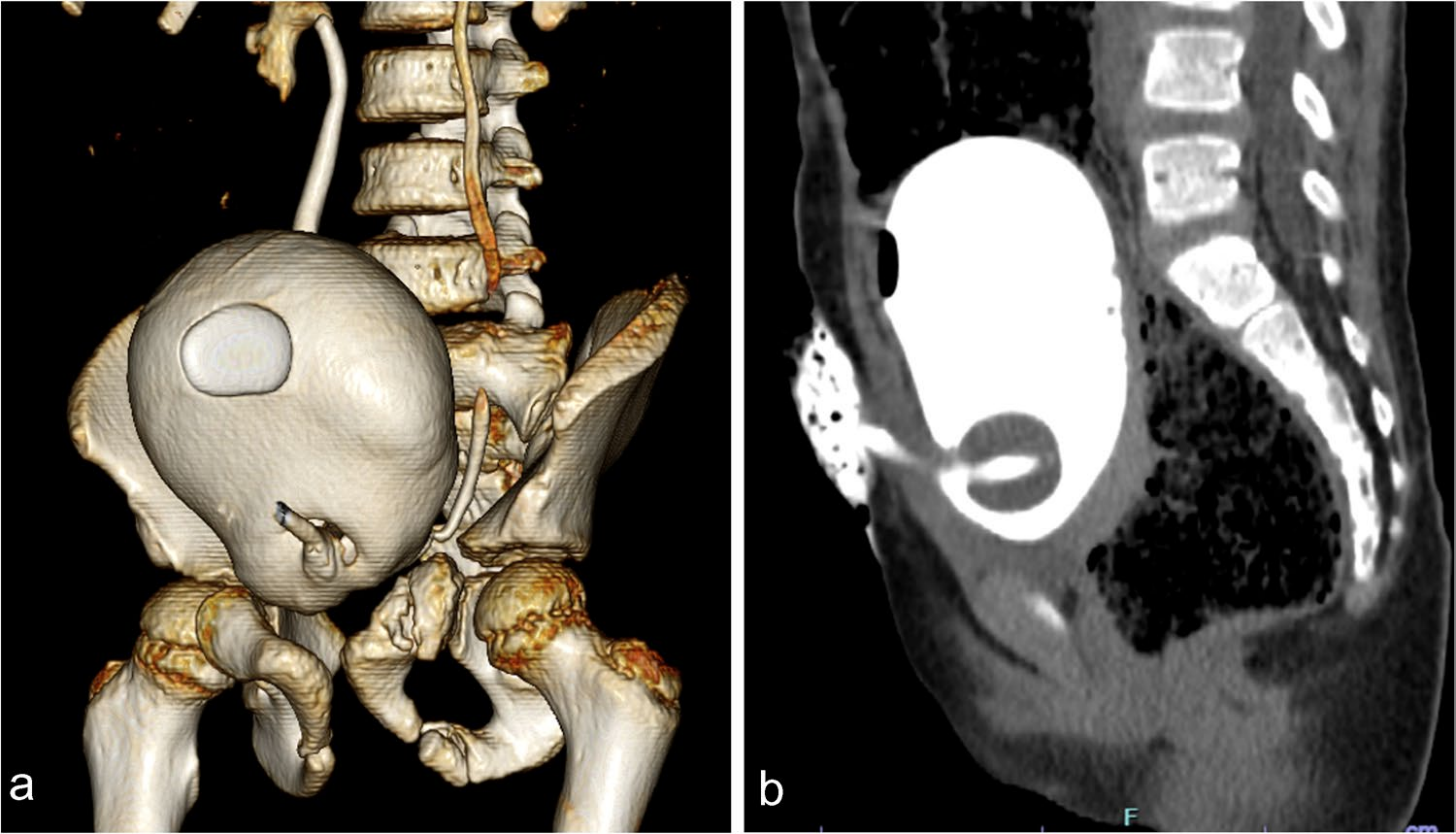
A 4-year-old girl with a fractured pelvis and complete urethral transection. **a**, **b** Computed tomography cystograms with three-dimensional reconstruction (**a**) and sagittal reconstruction (**b**) show full distension and opacification of the urinary bladder with no contrast extravasation. The patient is unable to micturate. Note the superior displacement of the urinary bladder

Urinary bladder (UB) rupture may be intraperitoneal or extraperitoneal. Extraperitoneal rupture usually occurs due to bone fragments associated with pelvic fracture, usually fracture of the obturator ring (Fig. 15), the sacrum, and diastasis of the sacroiliac joint. Intraperitoneal rupture is a lap belt-induced injury to a distended bladder. Bladder rupture can be determined by cystography following retrograde administration of contrast media. Cystography can be performed by either CT or fluoroscopy (Figs. 15 and 16). CT is more sensitive in the identification of extravasated contrast and more accurate in the determination of the pathway of leakage. This is important for differentiating intraperitoneal rupture from extraperitoneal rupture. Contrast agent leaks into the lateral perivesical and lateral pericolic spaces in intraperitoneal rupture, whereas in extraperitoneal rupture, contrast agent leaks superiorly into the umbilicus or in the presacral space [72–74]. Although trauma surgeons and urologists often rely on the AAST UB injury scale to categorize UB injuries, it is essential to recognize that its grading is based on surgical or endoscopic observations rather than imaging findings, incorporating size criteria for lacerations [40]. However, due to the bladder’s spherical shape, variations in distension during CT cystography, and the frequent difficulty in directly visualizing lacerations, imaging alone may not provide accurate measurements. Consequently, most institutions prefer using the radiologic classification system by Sandler et al. for grading bladder injuries on CT [75] (Supplementary Table 2).

## Pancreatic trauma

**Fig. 17:**
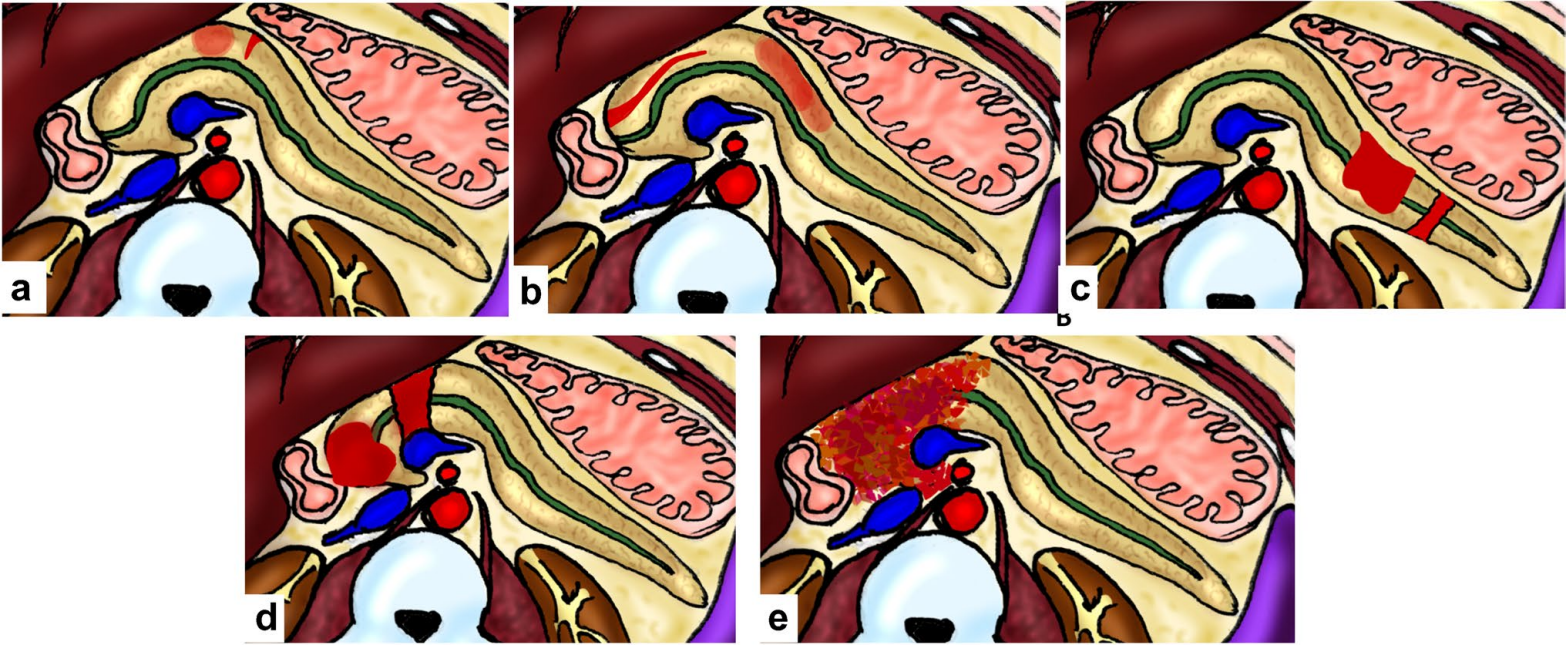
A diagram illustrating the different grades of pancreatic injury. **a** Grade I represents a small contusion or superficial laceration without duct injury. **b** Grade II injury includes large contusion or laceration without duct injury. **c** Grade III injury involves distal pancreatic transection or deep lacerations with distal duct injury. **d** Grade IV includes proximal pancreatic transection or deep injuries affecting the ampulla and/or intrapancreatic common bile duct. **e** Grade V includes massive parenchymal disruption of the pancreatic head (shattered pancreas)

Pancreatic trauma in children, although less common than in adults, poses significant diagnostic and management challenges. Children may present with nonspecific symptoms, making early recognition crucial [76]. Pancreatic injuries may be associated with hepatic, splenic, or duodenal injuries. Pancreatic injury may be difficult to identify due to its small size and paucity of surrounding fat, whereas undulation or folding of the normal pancreatic tissue may be mistaken for laceration [64]. Trauma may vary in severity from minor contusions to major lacerations or ductal injuries (Fig. 17) (Supplementary Table 1). The presence of fluid in the anterior pararenal space or lesser sac is the best indicator of pancreatic injury [76]. Other imaging signs include increased pancreatic size, peripancreatic fat stranding, free intraperitoneal fluid, and the formation of pseudocysts. These changes are secondary to trauma-induced pancreatitis. Pancreatic trauma can be complicated by splenic artery pseudoaneurysm [76].

**Fig. 18:**
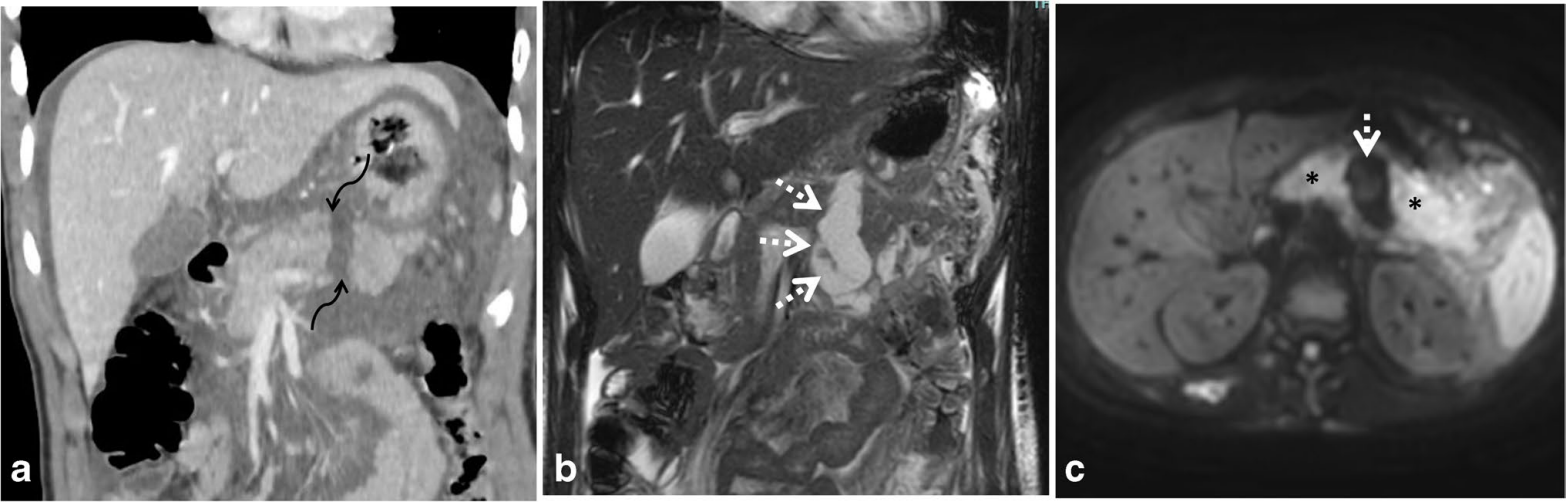
A 16-year-old boy with abdominal trauma and grade III pancreatic injury. **a** Coronal contrast-enhanced computed tomography shows distal hypodense laceration of the pancreatic body (transection), with a 10-mm fluid-filled gap (*curved arrows*) denoting pancreatic duct injury. **b** Coronal T2-weighted magnetic resonance imaging shows a localized fluid collection (*arrows*). **c** Axial diffusion-weighted image shows that the collection exhibits facilitated diffusion (*dotted arrow*). Note that the pancreas (*asterisk*) shows diffusion restriction denoting traumatic pancreatitis

Pancreatic duct injury has therapeutic and prognostic implications. Major pancreatic duct injury may be an indication for distal pancreatectomy. However, conservative management has been proven successful in most cases of pancreatic injury. Pancreatic duct injury can be predicted by CT when the laceration extends for more than 50% in the short axis, free intraperitoneal fluid, or loculated peripancreatic fluid. MRI and MRCP can be utilized as problem-solving tools in hemodynamically stable patients with suspected pancreatic duct injury if the initial CT scans are not conclusive (Fig. 18). Disruption of the continuity of the pancreatic duct and non-visualization at the site of injury represent signs of duct injury by MRI. MRCP can be acquired after the injection of secretin for better visualization of pancreatic duct injury and detection of leakage from the injured duct [32, 71]. MRI plays a crucial role in tracking the progression of pancreatic trauma over time. It effectively identifies delayed complications, including the formation of pseudocysts, pancreatic fistulae, and ductal strictures. Additionally, MRI can demonstrate direct communication between a pseudocyst and the pancreatic duct [69].

## Bowel injury

**Fig. 19:**
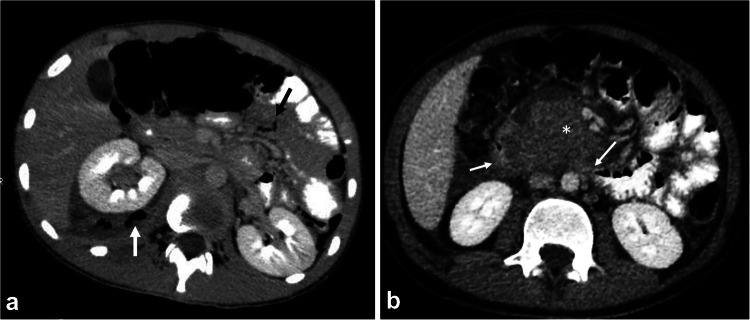
Axial contrast-enhanced computed tomography of two different patients with bowel injury. **a** A 14-year-old boy with duodenal perforation. There is a perinephric collection containing air bubbles (*white arrow*), along with pockets of free air observed between the bowel loops (*black arrow*)*.* **b** A 10-year-old boy with a hematoma (*asterisk*) involving the second and third parts of the duodenum (*arrows*) and extending to the mesentery

**Fig. 20:**
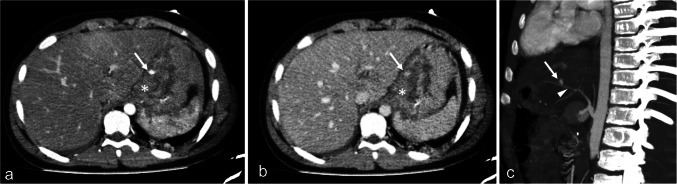
A 15-year-old boy with a gastric wall hematoma following a road traffic accident. **a-c** Axial contrast-enhanced computed tomography at the arterial (**a**) and venous (**b**) phases and oblique sagittal maximum intensity projection (**c**) show a gastric wall hematoma (*asterisk*) with contained vascular injury (*arrow*) following the blood pool in close proximity to the left gastric artery (*arrowhead*)

Although rare in children, bowel injury may occur due to motor vehicle accidents. The jejunum is the most frequently injured bowel loop followed by the duodenum and stomach [3]. Partial thickness injury leads to intramural hematoma, whereas full thickness injury leads to bowel rupture. Intramural hematoma is relatively common in the duodenum and manifests as focal bowel wall thickening without the presence of extraluminal air or contrast extravasation (Figs. 19 and 20). Duodenal injuries are usually associated with bicycle accidents [66]. Bowel rupture usually occurs in the jejunum and can be predicted by the presence of an unexplained large amount of ascites or by bowel wall interruption. Extraluminal air is visible in only one-third to half of cases (Fig. 19). A polygonal fluid collection in the mesentery near a focally thickened bowel loop should raise suspicion for bowel injury and potential perforation. Streaky infiltration of mesenteric fat, when accompanied by bowel wall thickening, is a strong indicator of severe bowel injury as well [66]. Mesenteric hematoma without bowel wall abnormalities is typically linked to mesenteric vessel damage [77].

## Conclusion

 Contrast-enhanced CT plays a vital role in evaluating blunt abdominal trauma in children and adolescents. Optimizing pediatric CT protocols and implementing a systematic approach to image interpretation facilitate a structured and thorough assessment, thereby improving diagnostic accuracy in trauma patients while reducing radiation exposure. This approach can be particularly valuable for radiology residents and emergency healthcare providers. CT grading ranges from grade I, which refers to minor injuries, to grade V, which encompasses extensive lacerations, major vascular injuries, or avulsion. By implementing the AAST-OIS grading system, healthcare professionals communicate using a unified language, enhancing interprofessional collaboration for better healthcare outcomes.

## Supplementary Information

Below is the link to the electronic supplementary material.

ESM 1 (DOCX 16.1 KB)

ESM 2 (DOCX 14.7 KB)

## Data Availability

No datasets were generated or analysed during the current study.
