# Supplementary material for: Computed tomography of pediatric abdominal trauma: optimizing utilization and enhancing diagnostic interpretation
Source: Pediatr Radiol. 2025 Jul 18;55(10):2018–36. doi: 10.1007/s00247-025-06321-3 (PMC12513975; doi:10.1007/s00247-025-06321-3)
Supplement: Supplementary file 1 — (DOCX 16.1 KB) [file 247_2025_6321_MOESM1_ESM.docx]

Supplementary table 1: Grading of the spleen, liver, kidney and pancreas according to the Revised 2018 American association for the surgery of trauma organ injury scale [40]

| Organ |  | Grade I | Grade II | Grade III | Grade IV | Grade V |
| --- | --- | --- | --- | --- | --- | --- |
| spleen | Subcapsular hematoma (surface area involvement) | <10% | 10-50% | >50% | vascular injury or active bleeding confined within splenic capsule  Parenchymal laceration involving segmental or  hilar vessels producing >25% devascularization | vascular injury or active bleeding extending to the peritoneum  Shattered spleen |
|  | Laceration depth | <1 cm | 1-3 cm depth | >3 cm |  |  |
|  | Intraparenchymal hematoma | ---------------- | <5 cm | ≥5 cm |  |  |
| Liver | Subcapsular hematoma | <10% | 10-50% | >50% ^a^ | Parenchymal disruption involving  25-75% of a hepatic lobe  Active bleeding extending beyond the  liver parenchyma into the peritoneum | Parenchymal disruption >75% of hepatic lobe  Retrohepatic vena cava and central major hepatic veins injury |
|  | Laceration | <1 cm depth | 1-3 cm depth  <10 cm length | >3 cm depth |  |  |
|  | Intraparenchymal hematoma |  | <10 cm | >10 cm  vascular injury or active bleeding  contained within liver parenchyma |  |  |
| Kidney | Subcapsular hematoma or contusion | Yes | Laceration ≤1 cm depth  without urinary extravasation  Perirenal hematoma confined to Gerota fascia | laceration >1 cm depth without  collecting system rupture or urinary extravasation  -Vascular injury  -Active bleeding contained within Gerota fascia | -laceration extending into  collecting system with urinary extravasation  -Renal pelvis laceration and/or complete  ureteropelvic disruption  -Segmental renal vein or artery injury  -Active bleeding beyond Gerota fascia into the  retroperitoneum or peritoneum  -Segmental or complete kidney infarction(s)  due to vessel thrombosis without active bleeding | Main renal artery or vein laceration or  avulsion of hilum  Devascularized kidney with active bleeding  Shattered kidney |
|  | Laceration | No |  |  |  |  |
| Pancreas | Hematoma | Minor contusion | Major contusion |  |  |  |
|  | Laceration | Superficial laceration | Major laceration | Distal transection | Proximal (to the patients’ right of the SMV) transection or parenchymal injury involving ampulla | Massive disruption of the pancreatic head |
|  | Duct injury | Negative | Negative | Distal duct injury |  |  |

a. ruptured capsule or parenchymal hematoma, *SMV* superior mesenteric vein
