# Supplementary material for: Computed tomography of pediatric abdominal trauma: optimizing utilization and enhancing diagnostic interpretation
Source: Pediatr Radiol. 2025 Jul 18;55(10):2018–36. doi: 10.1007/s00247-025-06321-3 (PMC12513975; doi:10.1007/s00247-025-06321-3)
Supplement: Supplementary file 2 — (DOCX 14.7 KB) [file 247_2025_6321_MOESM2_ESM.docx]

| AAST urinary Injury Scale [40] | | Radiologic classification system for traumatic injury [75] | |
| --- | --- | --- | --- |
| I | Intramural hematoma  Laceration, partial thickness tear | 1 | Urinary bladder contusion  Usually normal CT appearance, yet when detectable, it appears as mild focal wall thickening |
| II | Extraperitoneal bladder wall laceration less than 2 cm | 2 | Intraperitoneal rupture |
| III | Extraperitoneal (equals to or more than 2 cm) or intraperitoneal (less than 2cm) bladder wall laceration | 3 | Interstitial injury  It appears as wall irregularity related to intramural blood and contrast extension though a defect in the mucosa without transmural extension |
| IV | Intraperitoneal bladder wall laceration equals to or more than 2cm | 4 | Extraperitoneal rupture  4a: Simple: Contrast leakage confined to the extraperitoneal, intrapelvic space  4b: Complex: Contrast leakage into the anterior abdominal wall, penis, scrotum, and/or perineum |
| V | Intraperitoneal or extraperitoneal bladder wall laceration extending into the bladder neck or trigone | 5 | Combined intraperitoneal and extraperitoneal rupture |

Supplementary file: urinary bladder Injury Scale and Radiologic classification system for traumatic urinary bladder injury

*AAST* The American Association for the Surgery of Trauma
